# Cytological diagnostic features of late breast implant seromas: From reactive to anaplastic large cell lymphoma

**DOI:** 10.1371/journal.pone.0181097

**Published:** 2017-07-17

**Authors:** Arianna Di Napoli, Giuseppina Pepe, Enrico Giarnieri, Claudia Cippitelli, Adriana Bonifacino, Mauro Mattei, Maurizio Martelli, Carlo Falasca, Maria Christina Cox, Iolanda Santino, Maria Rosaria Giovagnoli

**Affiliations:** 1 Department of Clinical and Molecular Medicine, Sapienza University, Pathology Unit, Sant'Andrea Hospital, Roma, Italy; 2 Department of Clinical and Molecular Medicine, Sapienza University, Cytology Unit, Sant'Andrea Hospital, Roma, Italy; 3 Breast Unit, Sant’Andrea Hospital, Sapienza University, Roma, Italy; 4 Department of Cellular Biotechnologies and Hematology, Sapienza University, Policlinico Umberto I, Rome, Italy; 5 Hematology Unit, Sant’Andrea Hospital, Roma, Italy; 6 Department of Clinical and Molecular Medicine, Sapienza University, Microbiology Unit, Sant'Andrea Hospital, Roma, Italy; Universidad de Palermo, ITALY

## Abstract

Late breast implant seroma may be the presentation of a breast implant-associated anaplastic large cell lymphoma (BI-ALCL), which claims for a prompt recognition. However, BI-ALCL diagnosis on fine-needle aspiration (FNA) might be challenging for pathologists lacking experience with peri-implant breast effusions. Sixty-seven late breast implant seromas collected by FNA from 50 patients were evaluated by Papanicolaou smear stain and immunocytochemistry on cell blocks. A diagnostic algorithm based on the cellular composition, cell morphology and percentage of CD30^+^ cells was developed. Histological evaluation of the corresponding peri-prosthetic capsules was also performed. Most of the effusions (91% of the samples) were classified as reactive and 9% as BI-ALCL. In the BI-ALCL cases, medium-to-large atypical cells expressing CD30 represented more than 70% of the cellularity, whereas in in the reactive effusions CD30^+^ elements were extremely rare (<5%) and consisted of non-atypical elements. The reactive effusions were categorized into three patterns: i) *acute* infiltrate with prominent neutrophilic component (33% of the samples); ii) *mixed* infiltrate characterized by a variable number of neutrophils, lymphocytes and macrophages (30% of the samples); iii) *chronic* infiltrate composed predominantly of T lymphocytes or macrophages with only sporadic granulocytes (37% of the samples). The inflammatory cytological patterns were consistent with the histology of the corresponding capsules. Our results indicate that cytological analysis of late breast implant effusions, supported by the knowledge of the heterogeneous cytomorphological spectrum of late seromas, is a valuable approach for the early recognition of BI-ALCL.

## Introduction

Late peri-implant seroma is a complication of breast prosthetic reconstruction and mammoplasty [1]. Among the causes of late seroma development, infection (even subclinical), implant rupture, mechanical shearing, and breast implant-associated anaplastic large cell lymphoma (BI-ALCL), have been reported. BI-ALCL is a provisional entity recently included within the group of ALK-negative ALCLs [2]. It could manifest as a solid mass infiltrating the peri-prosthetic fibrotic capsule and soft tissues or, more frequently, as a late peri-implant seroma within which tumor cells are confined [3], [4]. It has been suggested that the effusion and the mass represent different stages of the same disease rather than two distinct clinicopathological variants and that they deserve different treatments [4], [5]. Since, the risk of lymph node involvement and of systemic spread depends on the invasion of the capsule, implant removal and total capsulectomy are indicated in non-invasive BI-ALCL, whereas systemic therapy is recommended by some authors in infiltrating tumors [2]. Therefore, cytological examination of the late peri-implant seroma emerges as a crucial procedure to clarify the nature of the effusion and to promptly diagnose BI-ALCL.

BI-ALCL also can be diagnosed on capsulectomy specimens. Nevertheless, the identification of localized BI-ALCL cases, in which scant tumor cell clusters adhere to the luminal surface of the capsule, might be challenging, particularly when a wide sampling of the capsule is not performed [6], [7], [8]. Flow cytometry (FC) may be a valuable tool for the diagnosis of BI-ALCL [9], [10]. However, in the routine diagnostic testing of late peri-implant breast seromas flow cytometry is restricted to adequately equipped institutions and to effusions with sufficient viable events. Moreover, as for the diagnosis of other large-cell lymphomas [11], flow cytometry studies on BI-ALCL highlighted several issues related to lymphomatous cells falling outside the typical lymphocyte region, to the marked loss of T cell antigens, and to the variable expression of myeloid markers [9], [10]. These aspects, altogether, may pose a diagnostic challenge and may give false-negative results when not integrated with the morphologic evaluation of the cells and clinical data, particularly in cases containing a low percentage of neoplastic cells. Hence, cytological evaluation of late peri-capsular seromas emerges as the gold standard for a prompt BI-ALCL diagnosis. Nevertheless, the diagnosis of BI-ALCL based on the cytopathological examination of the late effusion may be difficult for pathologists lacking experience with peri-implant breast effusions. Detailed descriptions of the differential cytological features of BI-ALCL, as compared with those characterizing non-neoplastic late effusions, have been only anecdotically reported [7], [8]. In fact, over the past decade, implant-related seromas were not routinely submitted to cytopathological evaluation. Yet, the knowledge of the cytological features characterizing non-neoplastic late seromas is relevant to both the diagnosis and the treatment choice. According to the type of the associated immune response, late seromas could be successfully treated with more than one approach including antibiotics, percutaneous drainage, or capsulectomy with implant replacement [1]. In particular, effusions rich in neutrophils are most likely related to infections and deserve antibiotic therapy, while effusions with foamy macrophages and multinucleated giant cells could suggest implant rupture and imply surgical treatment.

In this study we report the results of a comprehensive cytological and immunocytochemical analysis of a large series of late seromas associated with breast implants, consecutively collected over three years. The morphological spectrum of reactive and neoplastic seromas is detailed providing a diagnostic algorithm for the cytological diagnosis of BI-ALCL and the subclassification of the reactive effusions.

## Materials and methods

### Case collection

Sixty-seven late onset peri-implant seromas (> 6 months from last breast surgery) from 50 patients were collected by ultrasound-guided fine-needle aspiration (US-FNA) and consecutively analyzed at our Institution from 2013 to 2016. All the effusions were sampled by a 25 Gauge needle when more than 10ml of fluid were detected by US. Written informed consent was obtained from patients upon sample collection. The study was performed in accordance with the Declaration of Helsinki and approved by the Ethics Committee of Sant’Andrea Hospital/University “Sapienza” of Rome (EC n. 82 SA_2017).

### Cytological preparation

Seroma samples were centrifuged at 2500 rpm for 10 minutes. After removal of the supernatant, Papanicolaou-stained smears were prepared from the buffy coat of the cell deposit. On the smears a detailed evaluation of cytomorphological parameters including cellularity, size of atypical cells, nuclear details (presence of nucleoli, nuclear shape), cytoplasmic details (amount, presence of vacuoles), presence of mitoses, necrosis, apoptotic bodies, and the background appearances (serous, fibrinous, necrotic, hematic), was performed.

In addition to the preparation of conventional smears, fluids with an abundant cell deposit were subjected to cell block preparation by mixing the remaining cell sediment with 200μl of human plasma. Subsequently, 2 drops of thromboplastin (RecombiPlasTin 2G, Instrumentation Laboratory, Bredford, MA, USA) were added and mixed. The mixture was allowed to stand for 2 minutes. The resultant clot was placed in a cassette, fixed with buffered formalin and embedded in paraffin (FFPE). Cell blocks were sectioned at 2μm thickness and stained with Haematoxylin and Eosin.

### Microbiological culture

Twenty-one of the 61 reactive late seromas were sent for routine aerobic, anaerobic and fungal cultures. Samples were incubated in brain-heart infusion broth (BHI) and in fluid thioglycollate medium (FTM) (BD Biosciences, California, USA). Samples were also streaked on Mannitol salt agar (MSA), Chocolate agar (CA), MacConkey agar (MCA), Sabouraud dextrose agar (SAB), Columbia CNA Agar (CNA) and Columbia medium supplemented with 5% sheep blood (COS) plates using sterile disposable inoculation loops and incubated at 37°C overnight. After 24 hours of incubation, the plates were observed for the presence of bacterial colonies. If no growth was observed, the plates were incubated for an additional 24 hours at 37°C. The sample in which no bacterial growth was observed after 48 hours of incubation were recorded as negative.

### Surgical specimens

Capsulectomy specimens of the 5 BI-ALCL patients were obtained within a maximum of 16 days of the cytological diagnosis. In patient 2 the capsulectomy was not total; the residual capsule was surgically removed 1 month later. To allow comparison between the cytologic and histologic features, capsular specimens received within a maximum of 10 days from the cytological diagnosis of reactive effusion were also evaluated. These consisted of 11 specimens obtained from patients who underwent surgery for hematoma, capsular contracture, breast implant rupture or relapsed seroma.

### Immunohistochemistry and assessment of staining

Immunohistochemistry on cell block and FFPE capsular specimens was performed using an automated immunostainer (Dako, Glostrup, Denmark) with the following primary antibodies: CD30 (clone Ber-H2), CD3 (clone F7.2.38), CD4 (clone 4B12), CD8 (clone C8/144B), CD68 (clone PG1), CD15 (clone Car-b), Granzyme B (clone GrB-7), IRF-4 (clone MUM1) (Dako), CD25 (clone 4C9, Novocastra, Newcastle Upon Tyne, UK), PAX5 (clone SP34, Thermo Scientific, Waltham, USA). Paraffin sections were pretreated using EnVision^™^ FLEX Target Retrieval Solution (Dako) and incubated with an optimal dilution of the primary antibody. The reaction was visualized with the EnVision Detection Kit (Dako) using 3–3’-diaminobenzidine chromogenic substrate. Sections were counterstained with EnVision FLEX Hematoxylin (Link) (Dako).

For every marker, positive cells were counted out of 10 non-overlapping randomly selected high-power microscopic fields (HPFs, 40×10), and the mean number of positively stained cells per HPF was recorded. The percentage of positive cells was calculated as the ratio between the mean number of stained cells per HPF and the mean number of total cells per HPF.

### Statistical methods

Student’s t-test for unpaired samples was used to compare the mean number of positive cells for CD30, CD3, CD68, CD4, CD8, PAX5 and polymophonucleates (PMN) among the three different types of reactive effusion (acute, chronic, mixed). *P*-values <0.05 were assumed as statistically significant.

### Clonality analysis of T-cell receptor gamma (*TRG*) gene

Molecular evaluation of the rearrangements of the gamma chain of the T-cell receptor (TCR) was performed based on to the BIOMED-2 protocol using *TRG* rearrangements molecular analysis kit (Master Diagnostica, Granada, Spain). The PCR amplification products were analyzed on a 3130 Genetic Analyzer (Applied Biosystems, Foster City, California) using the GeneMapper software. Results were interpreted according to the guidelines for interpretation of Ig/TCR clonality testing [12], [13] and in line with our previous experiences [14], [15]. The assay was performed on the DNA extracted using the QIAamp DNA Mini kit (Qiagen, Hilden, Germany) from FFPE sections of the capsules of the 5 BI-ALCL cases and of the cell block of one reactive seroma enriched in CD30+ elements.

## Results

### Clinical findings

**Fig 1:**
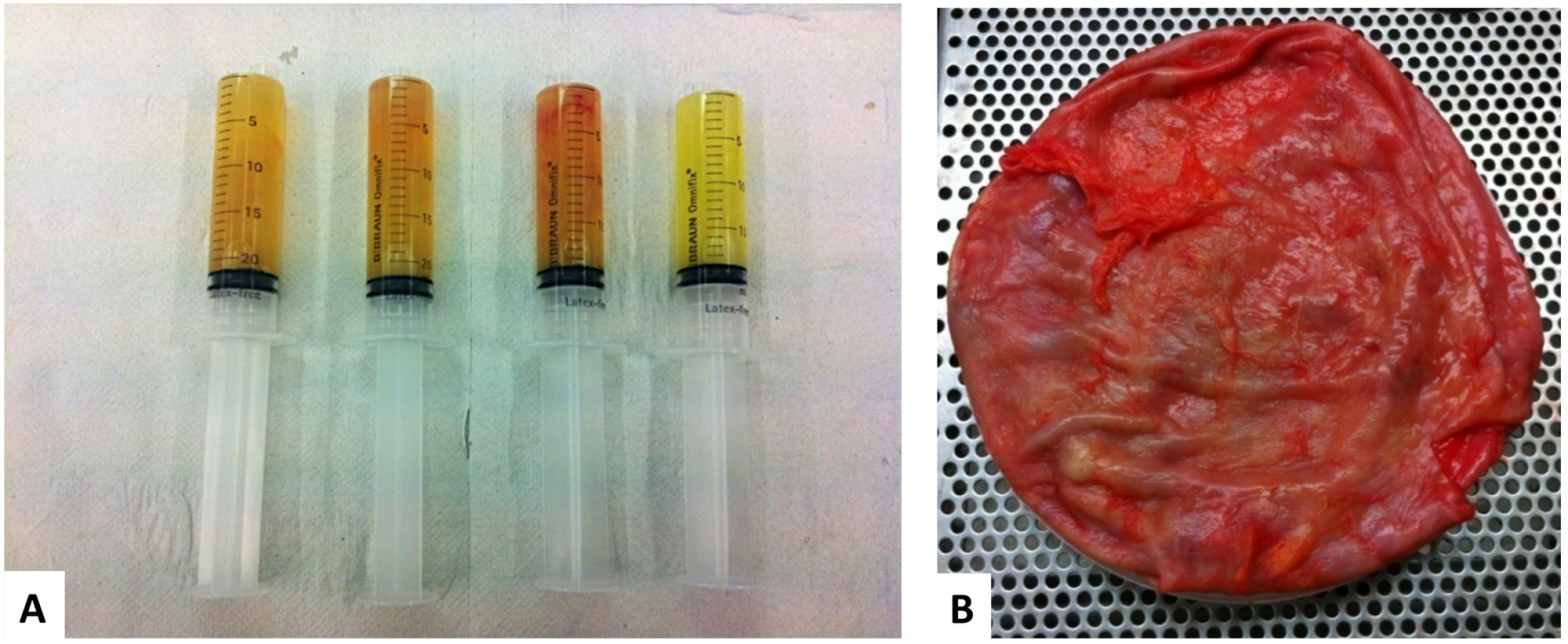
Late peri-implant breast seroma and total capsulectomy specimen of a patient with BI-ALCL. A. Ultrasound-guided fine-needle aspiration of 100mL of left-sided breast peri-implant fluid collection. The color of the seroma ranged from cloudy yellow to blood-stained. B. Grossly normal-appearing peri-prosthetic capsule with no evidence of solid or infiltrating masses.

Seroma specimens’ volume ranged from 20 to 600 mL and the color ranged from cloudy yellow to hemorrhagic (Fig 1A). Of the 67 late breast peri-mplant effusions collected from 50 patients, 65 were unilateral and 2 were bilateral. Thirty-eight out of 47 patients (81%) received breast implant after breast carcinoma surgery, 9 patients (19%) received implants for cosmetic reasons, while in 3 patients no clinical history was available. Forty-five out of 50 patients (90%) had a reactive seroma and 5 patients (10%) were diagnosed with BI-ALCL. All the patients were female, with a mean age at diagnosis of 51 years (range 35–72) for the BI-ALCL and of 53 years (range 23–75) for the reactive seroma patients. In the BI-ALCL group, the average time to seroma presentation from implantation or last surgery was 70 months (range, 48–96), whereas in the reactive group the average time to seroma development was 60 months (range, 6–137).

Based on the pathological staging of BI-ALCL proposed by Clemens and Colleagues [4], the 5 patients with BI-ALCL had lymphoma confined to the fibrous capsule with no invasion of the peri-capsular soft tissues and/or breast parenchyma. Involvement of controlateral axillary lymph nodes was present in one case (patient 1) at the time of diagnosis. All the BI-ALCL patients but one were treated with surgery alone (capsulectomy and implant removal); the patient with lymph node involvement received surgery and adjuvant chemotherapy consisting of 3 cycles of CHOEP (cyclophosphamide, hydroxydaunorubicin, vincristine, etoposide, prednisone) [16] and achieved complete remission. The mean clinical follow-up time of BI-ALCL patients was 36 months (range, 20–48). One of the five patients (patient 2) who had undergone partial capsulectomy experienced local recurrence during follow-up; accordingly, surgical removal of the residual fibrous capsule was performed. All the patients were disease-free at the time of last follow-up in December 2016. The clinical and pathological features of the BI-ALCL cases and of the reactive seromas are detailed in Table 1 and summarized in Table 2 respectively.

**Table 1:** Clinical and pathological features of BI-ALCL samples.

	Case 1	Case 2	Case 3	Case 4	Case 5
**Age at diagnosis (years)**	47	54	72	49	35
**Time interval between implant/last surgery and late seroma (months)**	72	60	72	96	48
**Side of seroma**	Left	Right	Right	Left	Left
**Reason for implant**	Reconstruction	Reconstruction	Cosmetic	Reconstruction	Cosmetic
**Breast carcinoma**	Left-sided infiltrating ductal breast carcinoma	Right-sided infiltrating ductal breast carcinoma	-	Left-sided infiltrating lobular breast carcinoma	-
**Type of implant**	BIOCELL^®^ textured silicone gel-filled (Inamed); contralateral breast augmentation with BIOCELL^®^ textured silicone gel-filled (Inamed)	BIOCELL^®^ textured silicone gel-filled (McGhan)	BIOCELL^®^ textured silicone gel-filled (Inamed) Replaced 8 years later with Polyurethane-coated silicone (Polytech Silimed)	BIOCELL^®^ textured silicone gel-filled (Inamed)	Polyurethane-coated silicone (Polytech Silimed)
**Treatment**	Implant removal, capsulectomy, CHOEP	Implant removal, capsulectomy	Implant removal, capsulectomy	Implant removal, capsulectomy	Implant removal, capsulectomy
**Follow-up (months)**	48	42	41	27	20
**Current status**	Disease-free	Disease-free	Disease-free	Disease-free	Disease-free
**Immunophenotype**	CD30+, ALK-, CD8-, GrB+/-, CD4+, CD3-, CD15+/-, IRF4+, CD25+	CD30+, ALK-, CD8-, GrB+/-, CD4+, CD3-, CD15+/-, IRF4+, CD25+	CD30+, ALK-, CD8+, GrB+,CD4-, CD3-, CD15-, IRF4+/-, CD25+	CD30+, ALK-, CD8-, GrB-, CD4+, CD3+/-, CD15+, PAX5-, IRF4+, CD25+/-	CD30+, ALK-, CD8-, GrB+/-, CD4-, CD3+/-, CD15-
***TRG* gene rearrangement**	Polyclonal	Monoclonal	Monoclonal	Monoclonal	Monoclonal

Adapted from Di Napoli A. et al (Ref. 18)

**Table 2:** Clinical, microbiological and pathological features of reactive late seromas.

**Clinical Features**	**No**.	**%**
No of patients	45	
Patients with recurrent seroma	8	18
Age at seroma presentation (years)		
Mean	53	
Range	23–75	
Time interval between implant/last surgery and late seroma (months)		
Mean	60	
Range	6–137	
Side		
Left	25	56
Right	18	40
Bilateral	2	4
Reason for implant		
Cosmetic	7	15
Reconstruction	35	78
n/a	3	7
**Cytological Features**	**No**.	**%**
N° of seromas	61	
Type of seroma		
Hemorrhagic/hypocellular/acellular	7	11
Cellular	54	89
Type of infiltrate		
Acute	18	33
Mixed	16	30
Chronic	20	37
**Microbiological culture**	**No**.	**%**
N° of seromas	21	
Type of infiltrate		
Acute	9	
non-sterile	4	44
sterile	5	56
Mixed	4	
non-sterile	1	25
sterile	3	75
Chronic	6	
non-sterile	0	0
sterile	6	100
Hemorragic	2	
non-sterile	0	0
sterile	2	100

### Cytological findings

Among the 67 effusions analyzed, 7 samples (10%) were hemorrhagic (3 cases), acellular (2 cases) or represented by scant foamy macrophages (2 cases), 54 samples (81%) were diagnosed as benign inflammatory infiltrates, and 6 samples (9%) as BI-ALCL.

**Fig 2:**
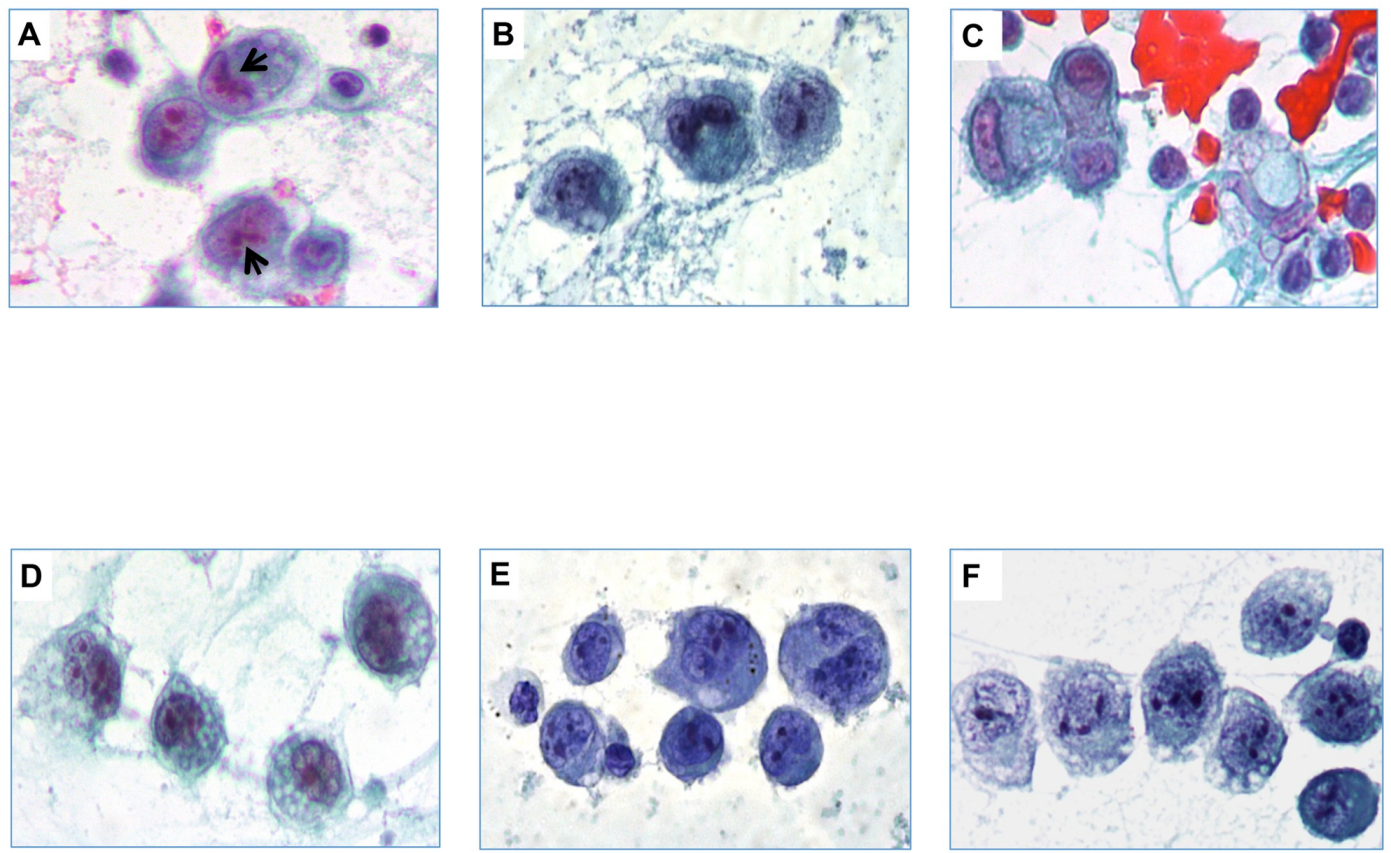
Aspirate smear of five cases of BI-ALCL. A. Case 1. B. Case 2. C. Relapse of case 2. D. Case 3. E. Case 4. F. Case 5. Smears showed large to medium-size atypical cells with irregularly shaped nuclei, conspicuous nucleoli and abundant clear cytoplasm in a fibrinous background. Morphologic features of BI-ALCL included hallmark cells with kidney-shaped nuclei (A arrows), binucleated Reed-Stenberg-like cells (B and E), cells with multiple nuclei (D) and mononucleated cells with prominent single or multiple nucleoli (D, E, F) (Papanicolaou smear, original magnification x400).

**Fig 3:**
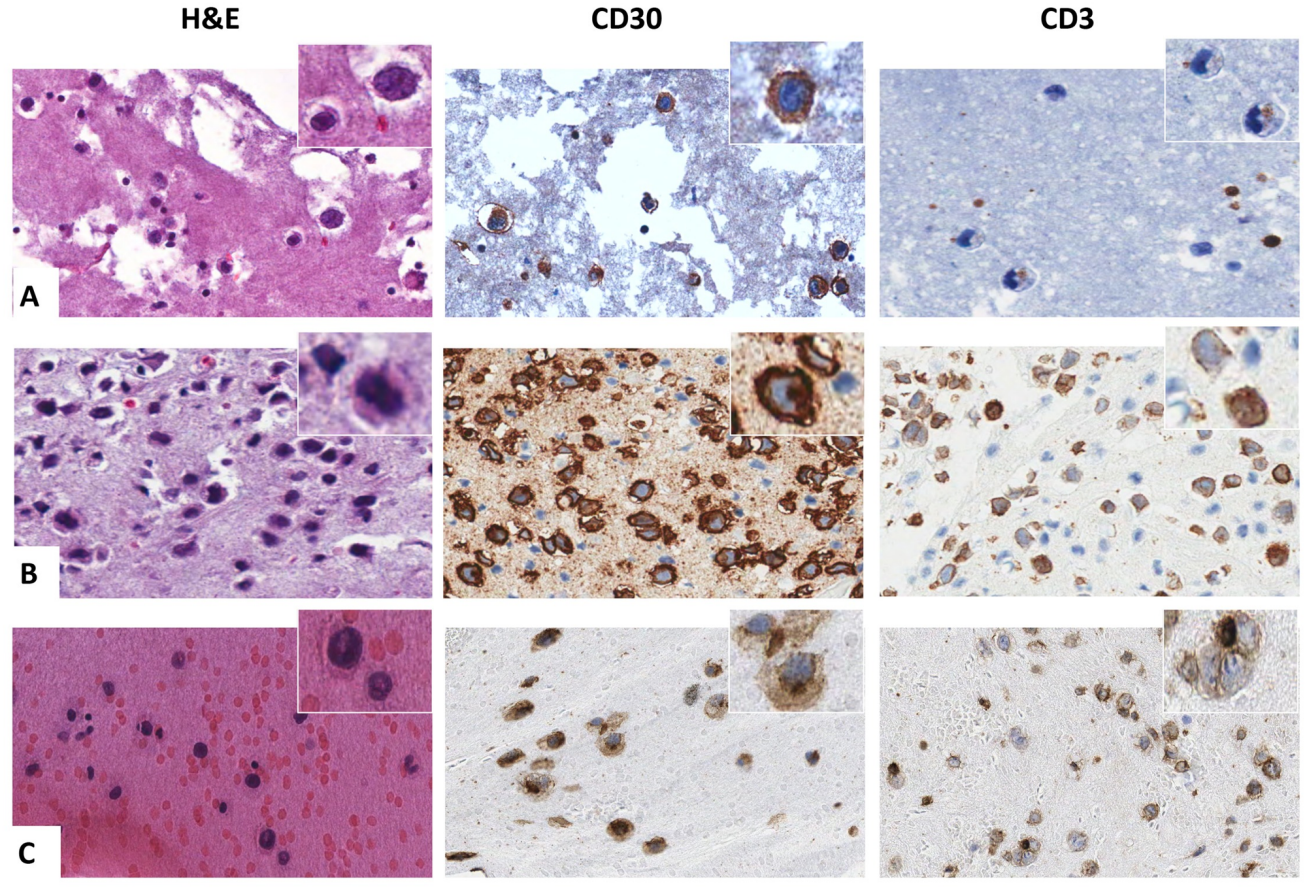
Immunophenotypic characterization of BI-ALCL cell blocks. Representative cases are shown to underline the variability of CD3 expression in contrast to the consistent, intense and diffuse positivity for CD30 in BI-ALCL. In A CD3 was negative in almost all the tumor cells. In B CD3 staining was heterogeneous with the lymphomatous cells being CD3-negative, CD3-weakly positive and CD3-strongly positive. In C the majority of the neoplastic cells showed a faint CD3 expression (all original magnification x400).

The smears of the neoplastic effusions showed large to medium-sized atypical cells (1.5-to-5 fold larger than a mature lymphocyte) with irregularly-shaped nuclei, prominent nucleoli and abundant clear and often vacuolated cytoplasm. The morphology of the neoplastic cells included hallmark cells with kidney-shaped nuclei, cells with multiple nuclei, binucleated Reed-Stenberg-like cells and mononucleated cells with prominent single or multiple nucleoli (Fig 2). Secondary features included the presence of a fibrinous background, apoptotic cells, and scattered atypical mitotic figures (<1%). In one sample eosinophils were also detected in the background. The cellularity varied from few large neoplastic cells admixed with small mature lymphocytes and macrophages to numerous large-to-medium sized atypical elements closely associated with small mature lymphocytes. In all the samples, atypical neoplastic cells were CD30 positive and accounted for about 70% of the total cellularity. The smear of the relapsed seroma, which occurred in one of the BI-ALCL patient, showed a mixture of lymphocytes and macrophages with scattered large atypical cells (Fig 2C) that upon CD30 immunostaining accounted for 10% of the total cellularity. Based on the presence of the CD30+ atypical cells and of the previous BI-ALCL diagnosis the seroma was intepreted as disease relapse. The immunophenotype of the BI-ALCL cases have been reported previously [17], [18] and is summarized in Table 1. All the cases showed a T-cell defective phenotype (Fig 3) with complete (case 1, 2, 3) or partial (case 4, 5) loss of CD3 expression. Most of the cases expressed CD4 except for case 3 that was CD8^+^ and for case 5 that expressed a CD4^-^ CD8^-^ double-negative phenotype.

**Fig 4:**
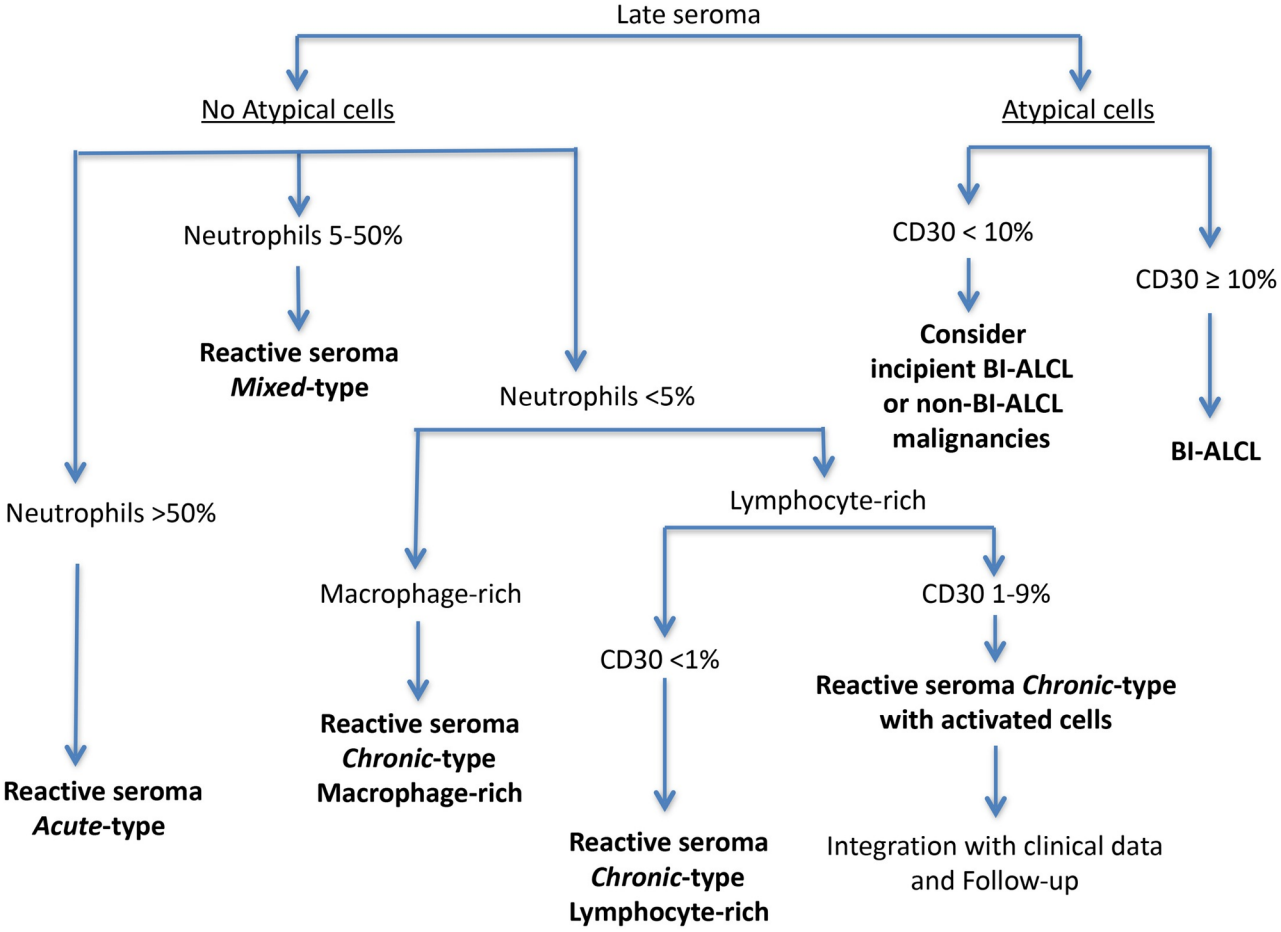
Algorithm for the cytological diagnosis of late peri-implant breast seromas based on morphology, cellular composition and CD30 immunostaining.

The cytological appearance of the reactive effusions was markedly heterogeneous among patients. Based on the cellular composition of the inflammatory infiltrate and the percentage of CD30^+^ elements, we developed a cytological diagnostic algorithm (Fig 4). The algorithm allowed the distinction of reactive seromas from BI-ALCL, and the classification of the reactive effusions into three main patterns: i) *acute*-type infiltrate with prominent polymorphonuclear granulocyte infiltration, mainly neutrophilic (>50% of the total cellularity), associated with some macrophages and scattered lymphocytes (18/54 samples, 33%); ii) *mixed-*type infiltrate characterized by a variable number of neutrophils (ranging from 5% to 50% of the total cellularity), admixed with lymphocytes and macrophages (16/54 samples, 30%); iii) *chronic*-type infiltrate composed by lymphocytes, macrophages and sporadic polymorphonuclear granulocytes, mainly eosinophils (<5% of the total cellularity) (20/54 samples, 37%) (Table 2 and Fig 4). In some of the *chronic*-type infiltrates (12 samples), the monocytic component, which included foamy macrophages and multinucleated giant cells, outnumbered the lymphocytes (macrophage-rich *chronic*-type infiltrates), while in other cases (8 samples) the lymphoid elements predominated over the monocytes (lymphocyte-rich *chronic*-type infiltrates) (Fig 4). Eight patients underwent multiple FNA along time: in 5 patients the pattern was unchanged, whereas in 2 cases the pattern switched from *mixed* to *chronic* and in one case from *mixed* to *acute*.

**Fig 5:**
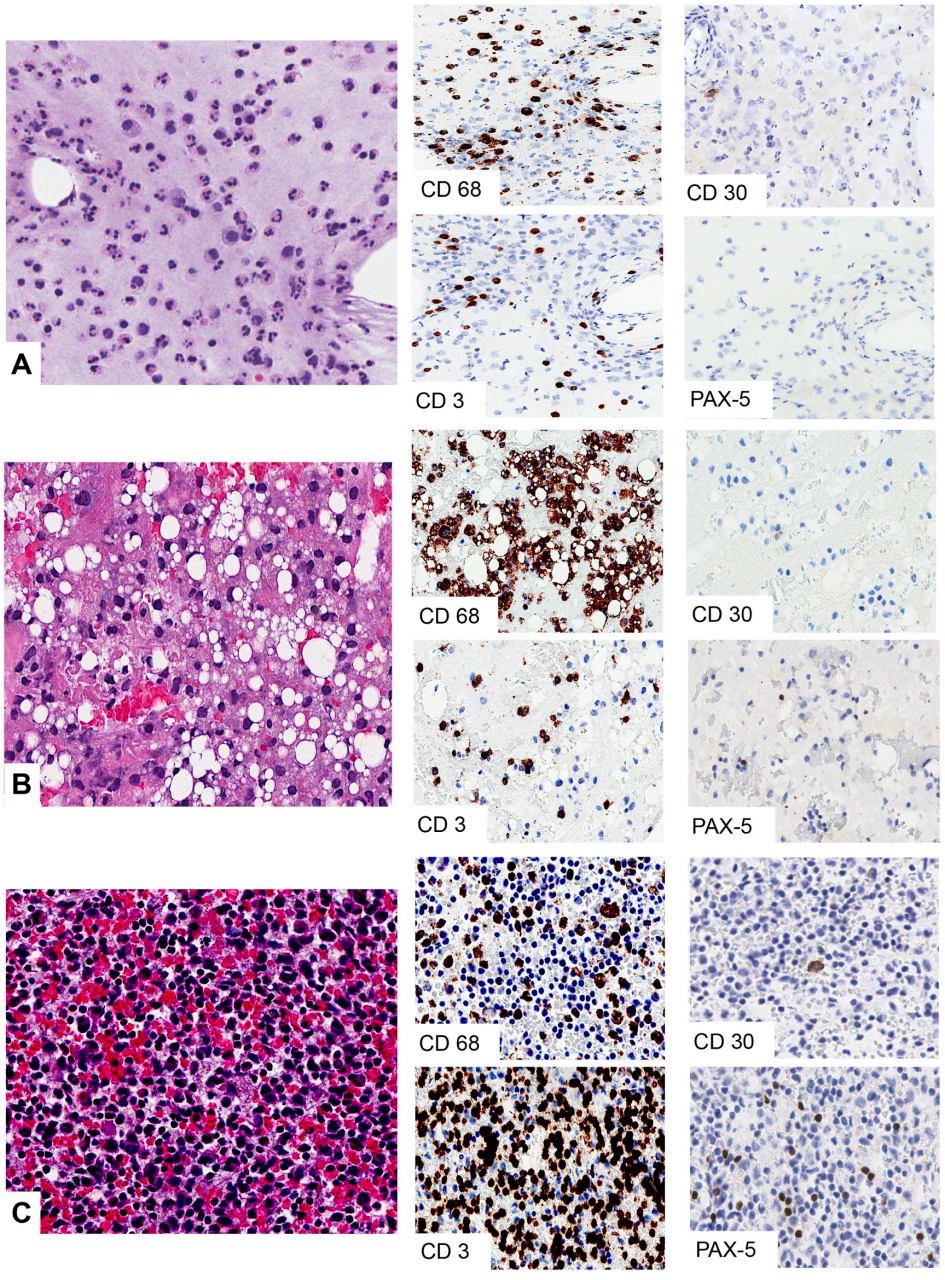
Cell block and immunohistochemistry of late onset breast peri-implant seromas. A. *Acute*-type reactive effusion with numerous polymorphonucleates admixed with CD68^+^ macrophages and scattered CD3^+^ T-cells. B. *Chronic*-type reactive effusion with predominance of foamy macrophages C. *Chronic*-type reactive effusion with predominance of T cells. In all the reactive effusions CD30^+^ cells were very rare or absent. Scant PAX5^+^ B-cells were detected in all types of seroma (H&E and immunohistochemistry original magnification x400).

Matched cell blocks were available in 21 reactive effusions. Based on the composition of the immune infiltrate cell block preparations were classified as follows: 6 *acute* infiltrates (neutrophils >55% of the total cellularity), 6 *mixed* infiltrates (neutrophils 8–54% of the total cellularity), and 9 *chronic* infiltrates (neutrophils <8% of the total cellularity) (Table 3 and Fig 5). In all the 21 cell blocks, the infiltrate pattern proved to be concordant with that observed on the corresponding smears. Immunocytochemical characterization showed a significant and progressive enrichment in CD68^+^ macrophages and in CD3^+^ T-cells moving from *acute* to *mixed* (CD68 *p* = 0.035; CD3 *p* = 0.0045) and from *acute* to *chronic* infiltrates (CD68 *p* = 0.0106; CD3 *p* = 0.0019) (Table 3). In the *acute* and *mixed* seromas, CD4^+^ lymphocytes outnumbered the CD8^+^ cells, whereas in the *chronic* effusions almost equal proportions of CD4^+^ and CD8^+^ T lymphocytes were recorded. B-cells were scant or completely absent in all the three patterns of reactive infiltrates, as confirmed by PAX5 immunostaining (Fig 5). Most importantly, cell blocks allowed for the immunohistochemical detection of cells expressing CD30 antigen, which, in the reactive effusion samples, were found with an average frequency below 1% of the overall cellularity (mean 0.4%, range 0–5). *Chronic* effusions showed the higher, although not statistically significant, percentage of CD30^+^ cells. (Table 3 and Fig 5).

**Table 3:** Immunophenotype of reactive late-onset breast peri-implant effusions.

Type of infiltrate	Cellularity	CD30 (%)	CD3 (%)	CD4 (%)*	CD8%)*	Pax5 (%)	CD68 (%)
**Chronic (n.9)**							
Mean±SD	393±291	1±1,64	40±24	56±19	44±19	3±4	54±26
Max	800	5	63	84	70	13	98
Min	80	0	2	30	16	0	27
**Mixed (n.6)**							
Mean±SD	483±475	0,15±0,30	31±19	65±11	35±10	1,74±1,79	45±22
Max	1200	1	53	77	50	5	75
Min	40	0	3	50	23	0	21
**Acute (n.6)**							
Mean±SD	475±399	0,02±0.02	6±2	66±17	34±17	0,27±0,5	23±15
Max	1100	0,05	10	88	50	1	41
Min	75	0	3	49	12	0	5
***p* value**							
Acute vs Mixed	0,4885	0,157	**0,0045**	0,4529	0,4529	**0,0416**	**0,0351**
Acute vs Chronic	0,3244	0,1524	**0,0019**	0,1827	0,1842	0,0589	**0,0106**
Chronic vs Mixed	0,3267	0,2004	0,2323	0,1646	0,1662	0,2094	0,2522

* relative to the number of CD3+ cells

**Fig 6:**
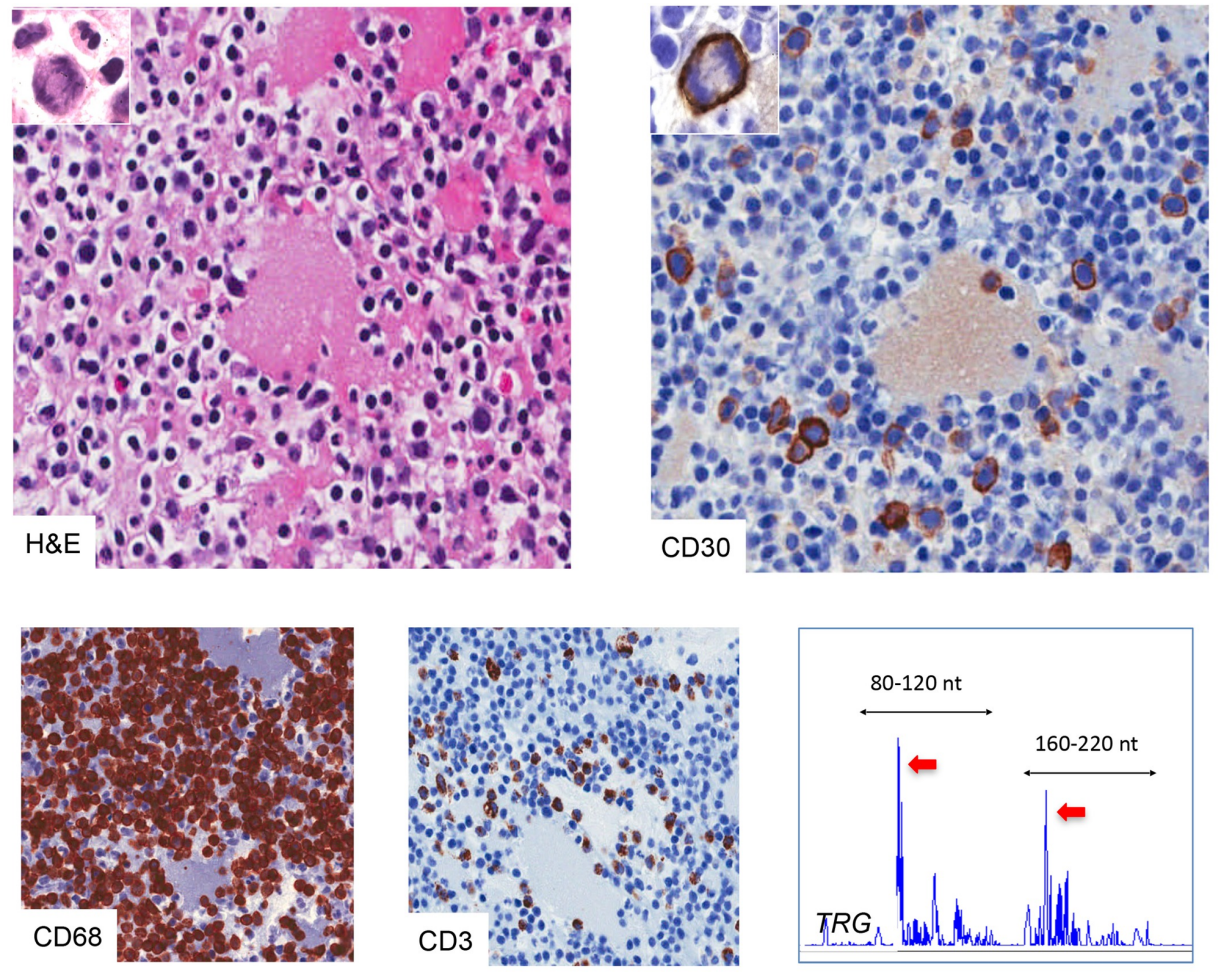
Morphological and molecular features of a CD30-rich reactive effusion. The seroma was composed mainly by CD3^+^ T cells associated with scattered CD68^+^ macrophages and polymorphonucleates. Immunohistochemistry for CD30 stained a fraction of medium-large proliferating cells equal to 5% of the total cellularity (H&E, original magnification x200; mitotic figures in the inserts x400). *TRG* (Vg 9/11—Jg) clonality by Genescan fragment analysis showed a prominent peak (red arrows) within a polyclonal background in both the reference size ranges given by the BIOMED2 protocol (black arrows).

Of note, the seroma of one of patient, a 32-years-old woman at the 8th week of gestation with a monolateral effusion occurring 11 years after breast augmentation, was classified as a *chronic*-type infiltrate and showed an unusually high fraction of CD30^+^ medium/large cells accounting for 5% of the overall cellularity (Fig 6). Mitotic figures involving large CD30^+^ elements were also detected. The *TRG* rearrangement analysis performed on the DNA extracted from the cell block, showed a predominant peak within a polyclonal background suggestive for a T-cell restricted expansion. Although the effusion was not associated with acute signs of inflammation, peripheral blood tests showed a mild neutrophilic leukocytosis (white blood cell count = 11,000/μL, neutrophil percentage = 93%) and an elevated C-reactive protein (CRP = 47mg/L). Based on these evidences a diagnosis of reactive seroma with excess of CD30^+^ blasts was made and an antibiotic therapy (Ampicillin, 250 mg orally two times a day for 10 days) was administered to the patient. In the 6 months following the diagnosis the patient was well and did not manifest any sign of recurrent seroma.

### Microbiological findings

Twenty-one out the 61 reactive late seromas were sent for culture. Based on cytological examination 9 effusions were classified as *acute*, 4 as *mixed*, 6 as *chronic* and 2 as hemorrhagic (Table 2). Culture was positive in 1 *mixed*-type seroma for both *Pseudomonas aeruginosa* and *Klebsiella oxytoca* and in 4 *acute*-type effusions for *Staphylococcus aureus* (3/4 cases) and for *Serratia marcescens* (1/4 cases). None of the 6 *chronic*-type seromas or of the 2 hemorragic effusions showed pathogen growth.

### Histological findings

**Fig 7:**
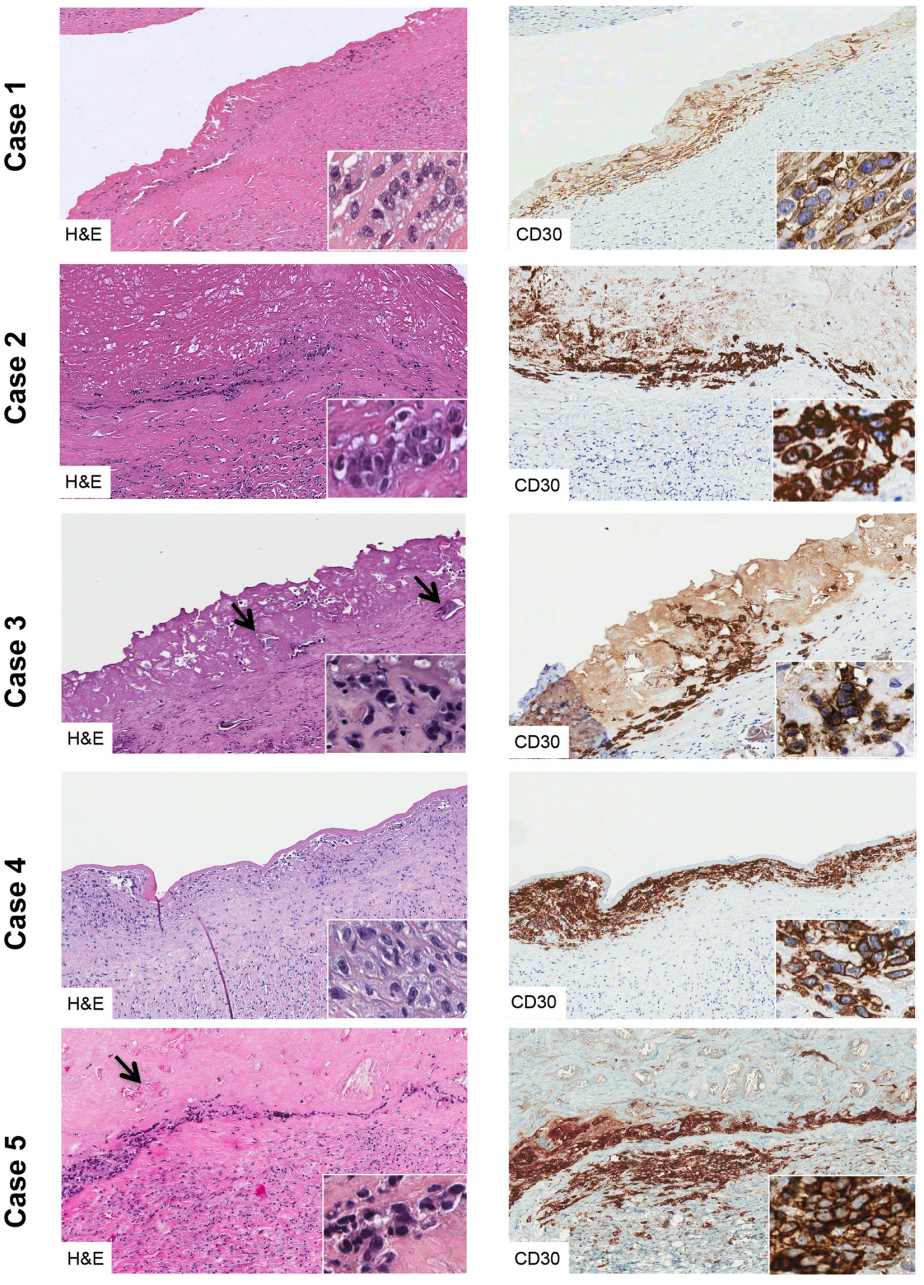
Histology of the 5 cases of BI-ALCL. In all the cases CD30-positive neoplastic cells were confined within the peri-prosthesis fibrous capsule. In case 3 and 5 tumor cells were smaller with a darker nuclei than case 1, 2, and 4. Small lymphocytes and histiocytes were sparse within the capsule. Arrows indicate polyurethane crystals in the fibrinous/necrotic material adherent to the luminal side of the capsule. (original magnification x 100, insert x400).

**Fig 8:**
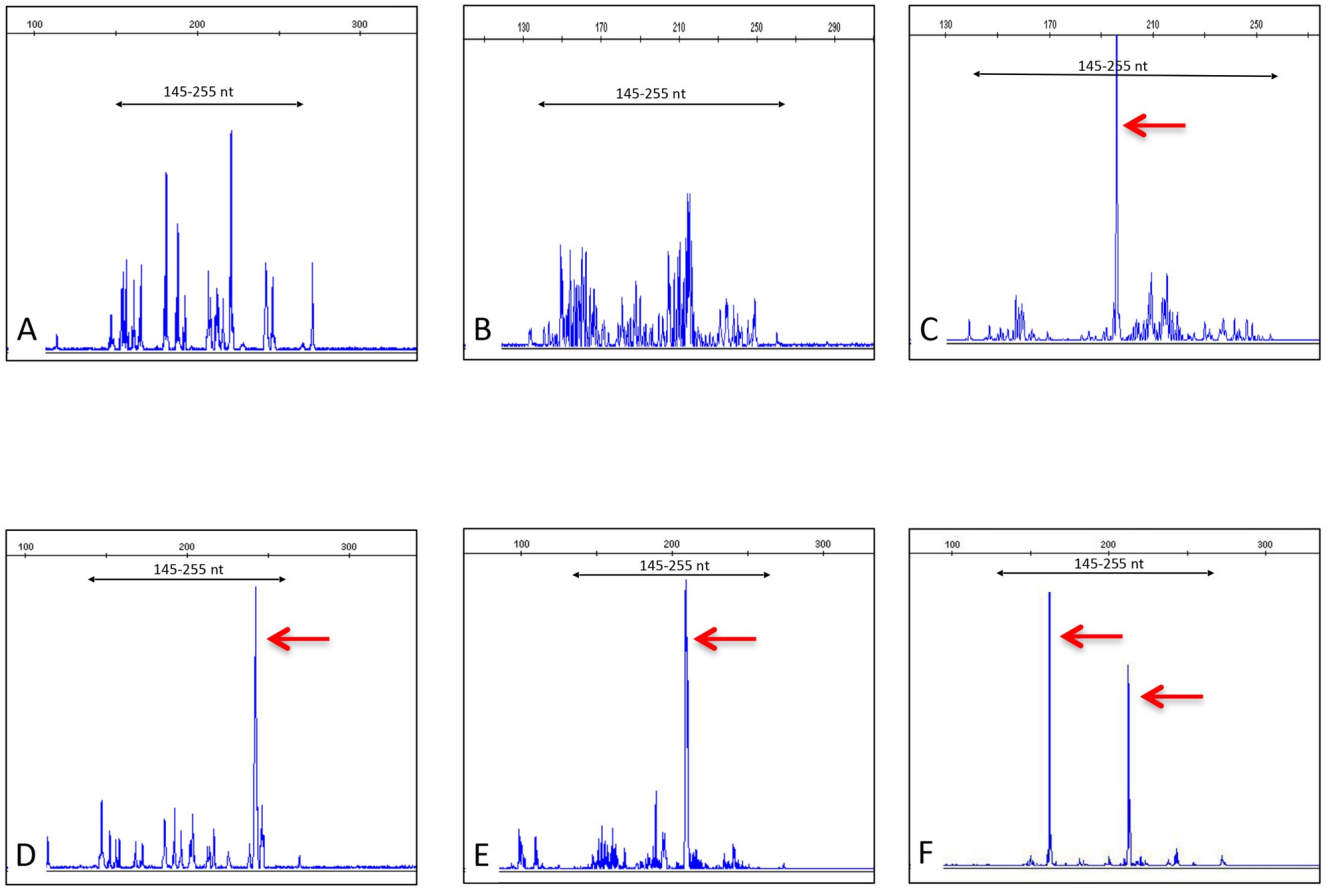
*TRG* (Vγ ^If/10^—Jγ) clonality by Genescan fragment analysis in BI-ALCL cases. An oligoclonal and a polyclonal pattern were observed in the capsule (A) and in the involved axillary lymph node (B) of case 1 respectively. Monoclonal rearrangement identified by a single high peak was found in case 2 (C), case 3 (D), and case 4 (E). Case 5 (F) showed a monoclonal biallelic rearrangement.

The five patients with a cytological diagnosis of BI-ALCL underwent implant removal and capsulectomy. Macroscopically, capsular specimens didn’t show the presence of solid masses (Fig 1B). Histological examination of the capsules confirmed the cytological diagnosis of BI-ALCL (Fig 7). In all the cases clusters of medium to large size cells with irregularly shaped nuclei and prominent nucleoli were found adherent to and within the luminal side of the fibrous capsule, whereas small lymphocytes and histiocytes were dispersed throughout the remaining layers of the capsule. Involvement of the contralateral axillary lymph node by CD30^+^ lymphomatous cells clustered within the sinuses (metastatic-like pattern), was detected in case 1. In this case, clonality analysis of the T-cell receptor gamma gene (*TRG*) performed on the DNA extracted from both the peri-implant capsule and the involved lymph node (Fig 8A and 8B) failed to identify a monoclonal peak. All the other BI-ALCL cases showed a monoclonal *TRG* rearrangement (Fig 8C, 8D, 8E and 8F).

**Fig 9:**
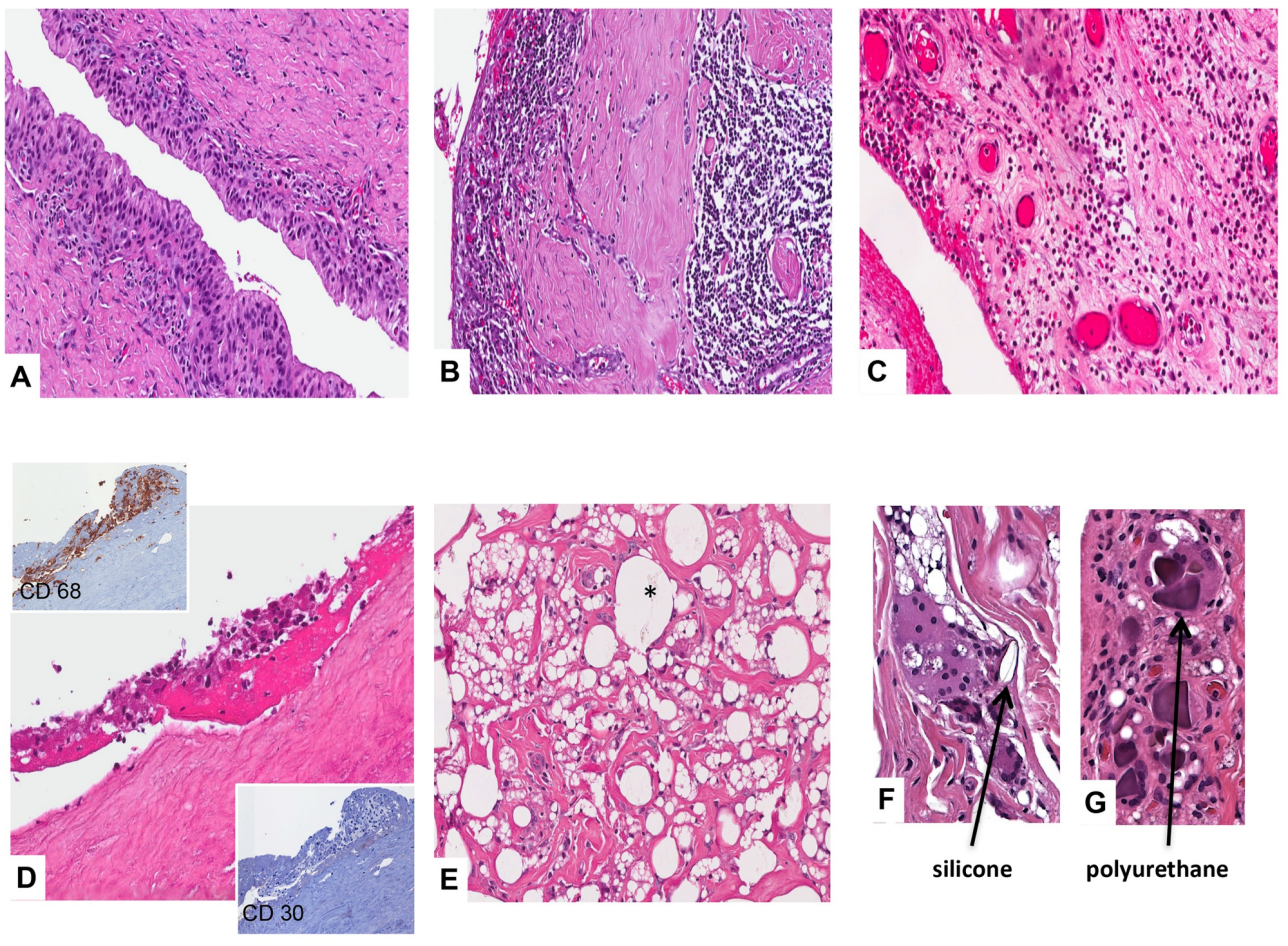
Histology of breast peri-implant fibrous capsules in patients with late-onset non-neoplastic seroma. A. Synovial metaplasia of the luminal side of the capsule associated with mild lymphocitic infiltrate. B. Intense chronic inflammation with numerous lymphocytes and plasmacells. C. Acute inflammation with neutrophils, edema and ectatic vessels. D. Sclerotic and acellular capsule with luminal aggregates of CD68^+^ CD30^-^ histiocytes. E. Diffuse histiocytic reaction with foamy macrophages and pseudo-cystic spaces containing gel-like refractile and color-less material compatible with silicone (asterisk). F and G. Silicone and polyurethan crystals within phagocyte vacuoles of multinucleated giant cells (original magnification H&E, CD30 and CD68 staining x200; F and G x400).

In 11 of the patients diagnosed with reactive effusions (4 *acute*, 3 *mixed* and 4 *chronic*) capsulectomy performed for breast implant revision at the time of FNA allowed comparison of the cytologic and histologic findings. In all the cases, the histology of the capsule was consistent with the cytology of the corresponding effusion (Fig 9). In particular, those patients with *acute* pattern seromas showed a neutrophilic inflammatory infiltrate involving the peri-prosthetic capsule and forming a granulation tissue (Fig 9C), whereas the *mixed* and the *chronic* effusions corresponded to a mononuclear chronic inflammation of the capsule, which in some cases was associated with the occurrence of synovial metaplasia (Fig 9A). In cases with synovial metaplasia the degree of the associated lymphoid infiltration was milder than that of cases lacking the metaplastic reaction (Fig 9B). A diffuse histiocytic reaction characterized by the presence of foamy macrophages, pseudo-cystic spaces, giant-cells and foreign body materials was also observed associated with *chronic* seromas (Fig 9E–9G). Of note, in one of these cases, aggregates of macrophages on the luminal layer of a sclerotic and acellular capsule mimicked the neoplastic cell clusters of BI-ALCL (Fig 9D). In this case, immunocytochemistry for CD68 and CD30 proved diriment.

## Discussion

BI-ALCL is a recently described lymphoma entity [2], which must be suspected in women with late-onset breast peri-implant effusions [19], [20]. To date there are no data on the incidence of BI-ALCL among patients with late seromas. The Food and Drug Administration (FDA) has received 359 medical device reports (MDRs) of BI-ALCL, including nine deaths, in the last six years [21]. Similarly, at May 2017 the registry created by the Italian Ministry of Health in 2015 recorded 81 BI-ALCL cases in Europe [22]. In our experience, BI-ALCL cases accounted for 9% of the unselected late peri-implant breast effusions, with most of them being confined to the capsule. This result underlines the importance of a systematic cytological screening of all late breast implant-related seromas.

As for other effusion-related lymphomas, cytological diagnosis of BI-ALCL might be challenging and may benefit from immunocytochemistry for CD30 [19], [23]. However, no guidelines for the morphological evaluation or tresholding of CD30^+^ cells in late seromas have been so far provided. CD30 expression is not specific of lymphomatous cells [24], [25] being also present on activated B and T lymphocytes, NK cells, monocytes and eosinophils [26], [27], [28]. Furthermore, CD30 expression can be induced on both T cells and B cells as a result of viral infection [29] or upon activation under autoimmune and chronic inflammatory conditions [30], [31], [32]. In almost all the reactive seromas we analyzed, CD30 identified small-to-medium-size non-atypical elements accounting for less than 1% of the total cellularity. On the contrary, in the neoplastic effusions CD30 stained the vast majority of the morphologically atypical cells. Given this clear difference the distinction between reactive and lymphomatous effusions might appear prosaic. Nevertheless, in the recurrent BI-ALCL seroma experienced by one of our patient CD30^+^ atypical cells did not exceed 10% of the total cellularity whereas, in the reactive seroma of the pregnant woman non-atypical proliferating CD30^+^ cells reached 5% of total cells. The morphology of the CD30^+^ elements together with the clinical data was crucial for establishing both diagnoses. Interestingly, in the latter case the higher number of CD30^+^ reactive cells might have related to the hormonal status of the patient, which might have boosted the reactive inflammatory response inducing CD30 expression on non-neoplastic T cells. The observation of Piccinni and Colleagues that Progesterone promotes both IL-4 production and membrane CD30 expression in established human Thl cell clones is an intriguing link to this hypothesis [33]. Furthermore, in this case *TRG* clonality analysis showed a prominent peak over a polyclonal background, consistent with an exaggerated reactive expansion of a restricted T-cell population, likely selected by an unknown antigen [11], [12]. Similarly, Kadin ME and collaborators have recently described a late seroma surrounding a breast implant in which flow cytometry detected an expanded population of CD30^+^/CD3^+^ T cells expressing TCRVβ13.2 in a polyclonal background [34]. Authors considered the seroma as the manifestation of a CD30^+^ lymphoproliferative disorder. However, it is not clear to us, which features favored this diagnosis, since authors reported the presence of “transformed lymphocytes” in the effusion. Conversely, lack of *TRG* monoclonality does not exclude malignancy. In one of our BI-ALCL cases *TRG* analysis did not show a monoclonal rearrangement in both the peri-implant capsule and the involved lymph node. We could not exclude the presence of a selective rearrangement of the beta chain (*TRB*), as reported in other ALCLs [35], or alternatively, the possibility that it might correspond to a ‘molecular null-ALCL’, as found in 24% of ALK-positive and in 11% of ALK-negative ALCLs [36]. These findings highlight the importance of collecting and integrating all clinical (e.g. signs of inflammation), laboratory (e.g. microbiology, blood test), morphological (i.e. cellular composition and percentage of atypical CD30^+^ cells) and molecular data (i.e. T-cell receptor clonality testing by genescan fragment analysis or flow cytometry) when diagnosing late seromas.

Most of the inflammatory effusions we analyzed were non-neoplastic in nature and cytologically nonspecific in terms of etiologic diagnosis. However, as with inflammatory responses in other sites, the cellular composition and the relative abundance of the different immune cell types may provide significant clues to the underlying cause. Neutrophilic inflammatory effusions may indicate a bacterial infection. There are several reports in the literature dealing with implant-related “early” and “late” infections [37], [38], [39], [40]. Late infections are generally considered as resulting from bacteremia and secondary colonization of prosthesis with bacteria entering the body at a distant site [40]. In textured implants *Staphylococcus aureus* and *Enterobacter* spp were found to be the most frequent microorganisms involved [38], [39]. Additionally, the biofilm, a sessile community of microorganisms adherent on the surface of the implants, has been related to antibiotic resistance in many subclinical infections and to the risk of capsular contracture and BI-ALCL development [41]. Similarly to Chai SM et al, who found that 3 out 6 reactive seromas were composed mainly of neutrophils associated with the presence of granular proteinaceous debris suggestive of an acute inflammatory process [8], we found an abundant neutrophilic infiltrate in 33% of the samples and bacterial growth in 44% of the *acute*-type seromas cultured.

On the contrary, sterile serum and serosanguineous peri-prosthesis collections have been associated with trauma causing breast implant rupture and bleeding from microfractures of rigid capsules [42], with the use of oral anticoagulant therapy [43], and with prolonged inflammatory reactions leading to endothelial injury and capillary permeability [44], [45]. Although no anamnestic data were available for most of our samples, we supposed that trauma, implant microrupture and silicone gel tearing were the most likely causative events of the effusions in which red blood cells and/or foamy macrophages and multinucleated giant cells were present. In keeping with this hypothesis, we and others [8] observed foreign material, compatible with silicone or polyurethan, within giant cells and macrophages in effusion smears and peri-implant capsules.

We might speculate that lymphocyte-rich *chronic* seromas represent the response to a viral infection or, alternatively, an exaggerated and persistent lymphoproliferation related to an underlining immune disorder of the host, which might predispose to BI-ALCL development. Molecular evidences of activating mutations of the JAK/STAT inflammatory pathway in BI-ALCL samples [18], [46] and of a higher (although not statistically significant) percentage of CD30^+^ cells detected in the *chronic* seromas support this hypothesis. Hence, among all seroma associated with breast implant, the lymphocyte-rich ones might represent those who deserve a more meticulous cytological evaluation (i.e. presence and percentage of atypical CD30+ cells, pattern of T-cell receptor rearrangement) and a careful follow-up to allow the identification of a possible malignant transformation of the CD30^+^ T-cell proliferation.

Lastly, the *mixed* and the *chronic*- type effusions might represent the temporal evolution of the *acute* form. Rheumatoid pleural effusion is an example of sterile exudative fluid in which multiple thoracenteses have showed transition from neutrophil-predominant to lymphocyte-predominant fluids [47]. In our experience, multiple FNA of late seromas showed that in the same patient the pattern of infiltrate can change or remain stable over time. This phenomenon could be intrinsically related to the causative agent or influenced by the therapy administered (i.e. antibiotics).

### Conclusions

Our study indicates that systematic cytological evaluation and CD30 immunostaining of all late peri-implant breast seromas allow early recognition of BI-ALCL and provide information about BI-ALCL incidence. The comprehensive cytological description of late seromas, integrated in the diagnostic algorithm reported here, is intended as a compendium for the cytopathologists in the prospect of an increasing number of breast effusions they will likely manage in daily practice. The application of the diagnostic algorithm to larger series of seromas will also offer the opportunity to validate or refine the proposed cut-off values in order to better identify those cases warranting closer follow-up. Definitely, ad-hoc designed studies focusing on the integration among anamnestic, imaging, laboratory, cytological and molecular data are still needed to clarify the etiology of late seromas and the relationship, if any, with specific patient’s characteristics, intrinsic implant feature, and the development of BI-ALCL.
